# Segmental-Dependent Solubility and Permeability as Key Factors Guiding Controlled Release Drug Product Development

**DOI:** 10.3390/pharmaceutics12030295

**Published:** 2020-03-24

**Authors:** Milica Markovic, Moran Zur, Noa Fine-Shamir, Ester Haimov, Isabel González-Álvarez, Arik Dahan

**Affiliations:** 1 Department of Clinical Pharmacology, School of Pharmacy, Faculty of Health Sciences, Ben-Gurion University of the Negev, Beer-Sheva 8410501, Israel; 2 Department of Pharmacokinetics and Pharmaceutical Technology, Miguel Hernandez University, 03550 San Juan de Alicante, Spain

**Keywords:** controlled release drug product, biopharmaceutics classification system, drug solubility, drug permeability, location-dependent absorption

## Abstract

The main factors influencing the absorption of orally administered drugs are solubility and permeability, which are location-dependent and may vary along the gastrointestinal tract (GIT). The purpose of this work was to investigate segmental-dependent intestinal absorption and its role in controlled-release (CR) drug product development. The solubility/dissolution and permeability of carvedilol (vs. metoprolol) were thoroughly studied, in vitro/in vivo (Octanol-buffer distribution coefficients (Log D), parallel artificial membrane permeability assay (PAMPA), rat intestinal perfusion), focusing on location-dependent effects. Carvedilol exhibits changing solubility in different conditions throughout the GIT, attributable to its zwitterionic nature. A biorelevant pH-dilution dissolution study for carvedilol immediate release (IR) vs. CR scenario elucidates that while the IR dose (25 mg) may dissolve in the GIT luminal conditions, higher doses used in CR products would precipitate if administered at once, highlighting the advantage of CR from the solubility/dissolution point of view. Likewise, segmental-dependent permeability was evident, with higher permeability of carvedilol vs. the low/high P_eff_ marker metoprolol throughout the GIT, confirming it as a biopharmaceutical classification system (BCS) class II drug. Theoretical analysis of relevant physicochemical properties confirmed these results as well. A CR product may shift the carvedilol’s solubility behavior from class II to I since only a small dose portion needs to be solubilized at a given time point. The permeability of carvedilol surpasses the threshold of metoprolol jejunal permeability throughout the entire GIT, including the colon, establishing it as a suitable candidate for CR product development. Altogether, this work may serve as an analysis model in the decision process of CR formulation development and may increase our biopharmaceutical understanding of a successful CR drug product.

## 1. Introduction

Oral drug absorption depends on various parameters: physicochemical (e.g., ionization, pKa, solubility, physicochemical stability, lipophilic nature, polar surface area (PSA), molecular weight,), physiological (e.g., gastrointestinal pH, surface area available for absorption, transit time, expression of certain transporters, enzymes), and parameters associated with the dosage form [1,2,3]. However, keeping this complexity in mind, it was determined that the drug permeability and solubility/dissolution in the gastrointestinal aqueous milieu are the two essential variables that guide absorption in the gastrointestinal tract (GIT) [4]. 

These two key factors, the solubility and the permeability, are location-dependent and can vary along the GIT. Change in pH or presence of bile salts can modify drug solubility/dissolution in a given intestinal segment; for a drug to be considered as a high solubility compound as per the biopharmaceutical classification system (BCS), it needs to be dissolved in an aqueous media (250 mL or less) with the different pH values relevant to the GIT lumen (1.0–6.8) [5,6,7]. Likewise, intestinal permeability is also location-dependent, and pertains in each region of the GIT [1,3,8,9]. Therefore, in different scenarios, e.g., drug discovery, drug and formulation development, and regulatory considerations, assigning the BSC class membership founded only on physicochemical drug features may lead to the incorrect decision [2,10,11,12,13]. Thus, regional-dependent permeability factors also need to be considered, for instance, expression of membrane transporters (influx/efflux) along the intestinal tract [11,14,15,16], luminal pH that influences the changes in the drug ionization [1,3,8,13], local water absorption [10], and others.

Segmental-dependent biopharmaceutical considerations are particularly important for controlled-release (CR) drug products; the drug is continuously released throughout the entire GI; therefore, it is not sufficient for a drug moiety to have suitable solubility/permeability in only one particular intestinal segment [17,18,19]. 

Carvedilol is a third-generation β-blocker, and is commonly used for treating hypertension, heart failure, and left ventricular dysfunction (LVD) [20,21]. The pharmacokinetics and pharmacodynamics of carvedilol from controlled release (CR) and immediate release (IR) products were compared in two clinical studies [22,23]. The data from these studies demonstrated that once-daily CR carvedilol is clinically correspondent to the IR carvedilol drug product administered two times a day, in patients with heart failure and asymptomatic post-myocardial infarction [23]. In addition, carvedilol CR maintains steady β_1_-adrenergic blockade with a dose administered once every 24 h [22]. Metoprolol is a passively transported drug which is not affected by the P-glycoprotein (P-gp) efflux transport [24], while carvedilol is a substrate of P-gp [25]. Carvedilol inhibits the activity of P-glycoprotein (P-gp) transporter [26,27]. It also undergoes extensive stereoselective first-pass metabolism; the main cytochrome P450 enzymes responsible for the metabolism of both R(+) and S(−)-carvedilol are CYP2D6 and CYP2C9, with some of the resulting metabolites having pharmacological activity. Despite its extensive first-pass metabolism, marketed carvedilol CR capsules have a bioavailability of 85% relative to IR tablets, with good clinical efficacy [23]. Hence, carvedilol was shown as a successful candidate for development as a controlled-release drug product, despite the fact that the pH variations along the GIT may significantly alter both the solubility and the permeability of this ionizable (basic) drug [28,29]. This raises the question of carvedilol’s location-dependent intestinal solubility and permeability and its successful use as a CR product. 

This work aimed to study the segmental-dependent biopharmaceutical consideration of carvedilol as a model basic drug, analyzed in view of CR scenario, allowing to pinpoint the rational for a successful CR drug product. Carvedilol solubility/dissolution and permeability were systematically investigated, through in vitro/in vivo (Octanol-buffer distribution coefficients (Log D), parallel artificial membrane permeability assay (PAMPA), rat intestinal perfusion) techniques, focusing on location-dependent variations, as well as theoretical physicochemical properties analysis of the drug. As a Food and Drug Administration (FDA) recommended standard for the low-high permeability class boundary, we used metoprolol, which also served as an accompanying model compound, since it is also marketed as a CR drug product. This study offers a deeper understanding of the factors that could influence segmental-dependent permeability and solubility in a controlled-release setting, and their contribution to a successful controlled-release drug product.

## 2. Materials and Methods

### 2.1. Materials

Carvedilol, metoprolol, sodium chloride, potassium phosphate monobasic, and sodium phosphate dibasic, hexadecane, octanol, and trifluoroacetic acid (TFA) were purchased from Sigma Chemical Co. (St. Louis, MO, USA). Water and acetonitrile (Merck KGaA, Darmstadt, Germany) were ultra-performance liquid chromatography (UPLC) purity grade. All other substances were of analytical reagent grade.

### 2.2. Solubility 

The pH-dependent solubility, as well as carvedilol solubility BCS classification, was evaluated by the shake-flask method, as previously reported [8,30]. Briefly, the equilibrium solubility of carvedilol was studied at 37 °C, at pH 7.5 with phosphate buffer (potassium phosphate monobasic and sodium phosphate dibasic), pH 4.5 acetate buffer (sodium acetate and acetic acid), and pH 1.0 maleate buffer (maleic acid). Five hundred microliters of buffer was added to glass vials, and excess carvedilol quantities were added to buffer-containing glass vials, until the solution was no longer clear. Equilibrium was verified by comparison of 48- and 72-h samples. The pH of each solution was measured following the drug’s addition to the buffer solution. The vial caps were firmly sealed, and the vials were placed in a shaking incubator (100 rpm, 37 °C). Before the drug concentration was analyzed, the vials were centrifuged at 10,000 rpm (10,621 rcf) for 10 min, and the supernatant was removed, followed by drug quantification with UPLC. For dose number (D_0_) calculations, the highest dose of carvedilol immediate-release (IR) oral drug product was taken to be 25 mg [22]. 

### 2.3. Octanol-Buffer Distribution Coefficients

Octanol-buffer distribution coefficients (Log D) for carvedilol and metoprolol were determined at pH 6.5, 7.0, and 7.5 using the shake-flask method [12,30]. This pH range represents the physiological pH relevant for the intestinal tract (naturally, permeability from the stomach is considered not significant). Carvedilol and metoprolol solutions were prepared in a phosphate buffer saturated with octanol (pH 6.5, 7.0, and 7.5.), and consequently equilibrated at 37 °C, 48 h with an equal volume of buffer saturated with octanol of corresponding pH. The aqueous and octanol phase were parted by centrifugation, and the concentration of the drug in the aqueous phase was quantified by UPLC; the drug in the octanol phase was determined by mass balance. 

### 2.4. Biorelevant pH-Dilution Dissolution Studies

An in vitro biorelevant pH-dilution dissolution study was performed (n = 5 each) as we have previously published [31,32,33], to simulate drug dose dissolution while traveling along the GIT, in two scenarios: carvedilol concentrations of 100 µg/mL vs. 320 µg/mL, simulating the highest IR dose (25 mg; COREG^TM^) and CR dose (80 mg; COREG CR^TM^) on the market, taken with 250 mL of water. An aqueous suspension of the drug dose was first diluted into HCl 0.1M to obtain a pH of 1.2 (dilution factor 1:0.66) and agitated for 15 min (100 rpm at 37 °C), to mimic the stomach compartment, as we have previously reported. Then, samples were further diluted with fasted state simulated intestinal fluid (FaSSIF) (Biorelevant.Com Ltd., London, UK) with a dilution factor (1:1) for 30 min, followed by a dilution factor of 1:1.5, agitated for 30 min, and consequently 2 other dilutions of 1:1 with agitation time of 1 h each, to closely mimic the conditions throughout the small intestinal travel; the complete time of the study was 3 hours and 15 min (with samples taken at time points 0, 15, 45, 75, 105, 135, 195 min). During the course of the study, samples were centrifuged, filtrated, and the drug concentration was instantly quantified by UPLC. The solubilized drug amount, quantified by UPLC, was compared to the total amount of drug, which was calculated using the initial drug dose and consequent dilutions. This comparison enabled evaluation of the fraction of dose dissolved vs. precipitated for the IR vs. CR simulated experiments. The pH gradient throughout the experiment was designed to mimic the physiological conditions along the GIT, with a final pH of 7.6.

### 2.5. Parallel Artificial Membrane Permeability Assay Studies 

In vitro permeability studies through an artificial membrane were carried out in the hexadecane-based parallel artificial membrane permeability assay (PAMPA) using Millipore (Danvers, MA) 96-well MultiScreen-Permeability filter plates with 0.3 cm^2^ polycarbonate filter support (0.45 μm). The filter supports in every well were impregnated with 15 μL of a 5% solution (*v/v*) of hexadecane in hexanes and were then permitted to dry for 1 h. This time frame allowed the hexanes to be entirely evaporated, producing a consistent hexadecane layer. The permeability studies using hexadecane layer were carried out according to the standard protocol, with minor modifications [13]. Briefly, both carvedilol and metoprolol solutions (n = 4) were prepared in phosphate buffer solution (pH 6.5, 7.0, and 7.5) with comparable ionic strength and osmolality (290 mOsm/L). PAMPA sandwich plates were composed of donor wells containing various drug solutions (200 μL), and the receiver wells containing blank buffers (300 μL). The plate was incubated at room temperature, and samples were taken from the receiver plates every hour for a total of 4 h. Apparent permeability coefficient (P_app_) was calculated from the linear plot of drug collected in the acceptor side vs. time with the following equation:

$$P_{\mathrm{app}}=\frac{\mathrm{dQ}/\mathrm{dT}}{A\times {\;C}_{0}}$$

where dQ/dt is the appearance rate in the steady-state of carvedilol/metoprolol from the receiver side, C_0_ is the starting drug concentration in the donor side (0.02 mM for carvedilol, and 0.1 mM for metoprolol), and A is the membrane surface area (0.048 cm^2^). Linear regression was used to acquire the steady-state appearance rate of the drug on the receiver side.

### 2.6. Rat Single-Pass Intestinal Perfusion (SPIP)

The rat effective permeability coefficient (P_eff_) of carvedilol and metoprolol in different intestinal regions was evaluated using the single-pass intestinal perfusion (SPIP) model. This experimental model was designed and validated to account for the complex physiological background of drug absorption along the GIT: the living animal, intact and viable GIT including tissue composition, membrane morphology, expression/distribution of functional transporters/enzymes, and the composition of the luminal milieu of the different segments, are all part of the high biorelevance of this model [34,35,36]. All animal experiments were performed according to the protocols accepted by the Ben-Gurion University of the Negev Animal Use and Care Committee (Protocol IL-07-01-2015). The animals (male Wistar rats weighing 230–260 g, Harlan, Israel) were housed and handled in agreement with the Ben-Gurion University of the Negev Unit for Laboratory Animal Medicine Guidelines. 

The experimental procedure used for the in situ experiments in rats was previously described [3,12,13,30]. Prior to the experiment, the rats were fasted overnight. Namely, rats were anesthetized and positioned on a 37 °C surface (Harvard Apparatus Inc., Holliston, MA, USA), and a 3 cm midline abdominal incision was performed. Considering the complexity behind each of the intestinal segments, permeability was simultaneously measured through 3 separate intestinal regions (length of 10 cm each); a proximal segment of the jejunum at pH 6.5 (beginning at 2 cm under the ligament of the Treitz), a distal segment of the ileum at pH 7.5 (finishing 2 cm above the cecum), and the colonic segment at pH 6.5 (approximately 6 cm); the pH values through each region corresponded to the physiological pH of that region [1,3]. Each intestinal segment was cannulated at both sides and was rinsed with the relevant blank perfusion buffer. Phosphate buffers containing carvedilol and metoprolol were prepared at pH 6.5 and 7.5, while maintaining similar ionic strength and osmolality (290 mOsm/L) in all buffers. All solutions were incubated in a water bath at 37 °C. The steady-state conditions were established by perfusing the drug-containing buffer solution (0.02 mM) for 1 h, and an additional hour of perfusion followed, with sample collection every 10 min. The pH value was determined in the outlet samples to ensure the there was no pH variation during the course of perfusion. At the end of the perfusion study, the drug concentration in the outlet samples was determined by UPLC, and the length of the intestinal segment used for perfusion was measured for further permeability calculations. The effective permeability (P_eff_; cm/s) through the gut wall was calculated through to the following equation:

$$P_{\mathrm{eff}}=\frac{-\mathrm{Qln}\;({C^{\prime }}_{\mathrm{out}}/{C^{\prime }}_{\mathrm{in}})}{2\pi \mathrm{RL}}$$

Q being the perfusion buffer flow rate (0.2 mL/min), ${C^{\prime }}_{\mathrm{out}}/{C^{\prime }}_{\mathrm{in}}$ is the ratio of the outlet and the inlet drug concentration that has been adjusted for water transport by the gravimetric method [37,38,39], R is the radius of the intestinal segment (conventionally used as 0.2 cm), and L is the length of the perfused intestinal segment.

### 2.7. Physicochemical Analysis

The theoretical fraction extracted into octanol (f_e_) was calculated using the following equation [40,41]:

$$f_{e}=\frac{f_{u}P}{1+f_{u}P}$$

where P stands for the octanol–water distribution coefficient of the unionized drug form and f_u_ is the drug fraction unionized at a certain pH. The f_u_ vs. pH was plotted according to the Henderson–Hasselbalch equation, using the following literature pKa values: 9.7 for metoprolol [42] and 7.8 for carvedilol [28].

### 2.8. Ultra-Performance Liquid Chromatography

An ultra-performance liquid chromatography (UPLC) instrument Waters (Milford, MA, USA) Acquity UPLC H-Class was equipped with a photodiode array detector and Empower software. The instantaneous determination of carvedilol and metoprolol was accomplished using a Waters Acquity UPLC XTerra C_18_ 3.5-μm 4.6 × 250 mm column. The gradient mobile phase consisted of 90:10 going to 30:70 (v/v) water:acetonitrile (containing 0.1% TFA) at a flow rate of 0.5 mL/min during 4 min. The wavelength of detection and retention times for carvedilol and metoprolol were 230 and 275 nm and 2.5, 3.1 min, respectively. UPLC injection volumes for all analyses were in a range from 2 to 50 μL. The limit of quantitation was termed as the lowest drug concentration that could be measured with an accuracy and precision of <20%, as per US Food and Drug Administration Guidelines. Precision was stated as the intra- and inter-day relative standard deviation (RSD). Intra-day accuracy and precision were determined by analyzing six replicates of control samples on the same day (samples of known concentration), while the inter-day accuracy and precision were evaluated by measuring six replicates of control samples on three different days. The carvedilol limit of quantification was 5 ng/mL, and for metoprolol 25 ng/mL, and the inter- and intra-day coefficients of variations were <1.0% and 0.5%, respectively.

### 2.9. Statistical Analysis

Log D studies and PAMPA assays were replicated with n = 6 and n = 4, respectively. Animal studies were replicated with n = 6. All values are stated as means ± standard deviation (SD). Statistically significant differences between the experimental groups were evaluated by the nonparametric Kruskal–Wallis test for multiple comparisons, and the two-tailed nonparametric Mann–Whitney *U* test for two-group comparison. A *p* < 0.05 was considered statistically significant.

## 3. Results

### 3.1. Solubility

The solubility data for carvedilol in the three pH values (1.0, 4.0, and 7.5) at 37 °C are presented in Table 1. The solubility data presents a complex picture: from pH 1.0 to 4.0, the solubility was rising, and again decreased towards pH 7.5, where the solubility was very low. The dose number was calculated using the subsequent equation: D_0_ = M/V_0_/C_s_, where M is the highest single-unit dose strength of carvedilol (25 mg) [22], V_0_ is the initial volume of water (250 mL), and C_s_ is the solubility at each pH; drug molecules with D_0_ ≤ 1 are considered highly soluble. At a pH of 1.0 and 7.5, the dose number for carvedilol was higher than 1, indicating low BCS solubility class membership. The chemical structure of carvedilol is presented in Table 2.

### 3.2. Biorelevant pH-Dilution Dissolution Studies

**Figure 1:**
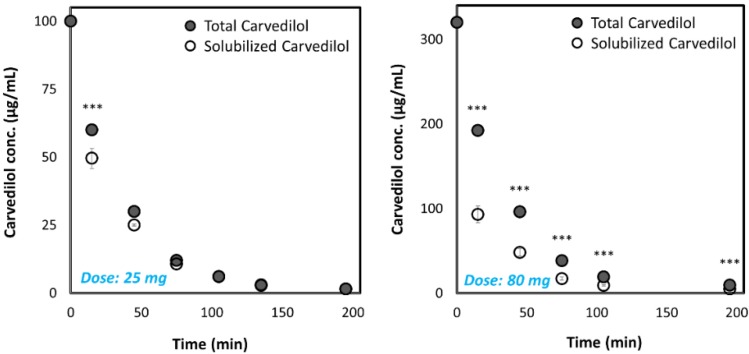
Dissolution of the highest carvedilol dose for IR and CR drug products on the market (25 mg and 80 mg, respectively) in the dynamic GIT environment, using the pH-dilution dissolution method. Values are presented as means ± SD; *** *p* < 0.001; n = 5. The pH at each time point for IR: 1.4 at 15 min; 1.9 at 45 min; 5.8 at 75 min; 6.9 at 105 min; 7.2 at 135 min; 7.3 at 195 min; and for CR drug product: 1.6 at 15 min; 2.9 at 45 min; 7.0 at 75 min; 7.4 at 105 min; 7.6 at 195 min.

We have studied the ability of the two highest marketed dosages for both IR (25 mg) and CR (80 mg) carvedilol drug products to accomplish and maintain complete dissolution of the carvedilol dose in the dynamic GIT environment using the pH-dilution method we have previously developed [31]. The dissolution results are presented in Figure 1, where it can be observed that a significant difference between the dissolution behavior of the 25 mg and 80 mg drug product was detected. The results indicate what may happen if these doses were to be orally administered at once; while the 80 mg dose would quickly precipitate, the 25 mg dose was completely dissolved (with ~15 min delay) and maintained dissolved throughout the GIT travel. 

### 3.3. Log D 

**Figure 2:**
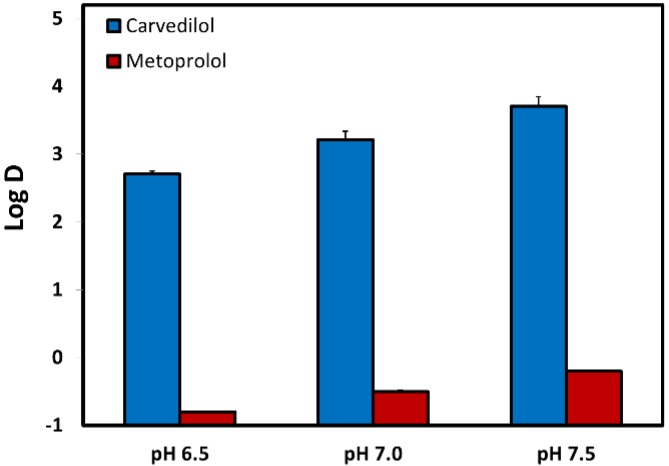
The octanol-buffer distribution coefficients for carvedilol vs. metoprolol at pH values of 6.5, 7.0, and 7.5. Values are presented as means ± SD; n = 6.

The octanol–water distribution coefficients (Log D) for carvedilol and metoprolol were measured at the three pH values of 6.5, 7.0, and 7.5, representative of the environment of the small intestine (Figure 2). It can be seen that both carvedilol and metoprolol have evident pH-dependent upward Log D in the investigated pH range (6.5–7.5), however, while the Log D of metoprolol ranged from 0.8 (pH 6.5) to −0.2 (pH 7.5), carvedilol Log D was positive and ranged from 2.7 (pH 6.5) to 3.7 (pH 7.5). 

### 3.4. Physicochemical Analysis

**Figure 3:**
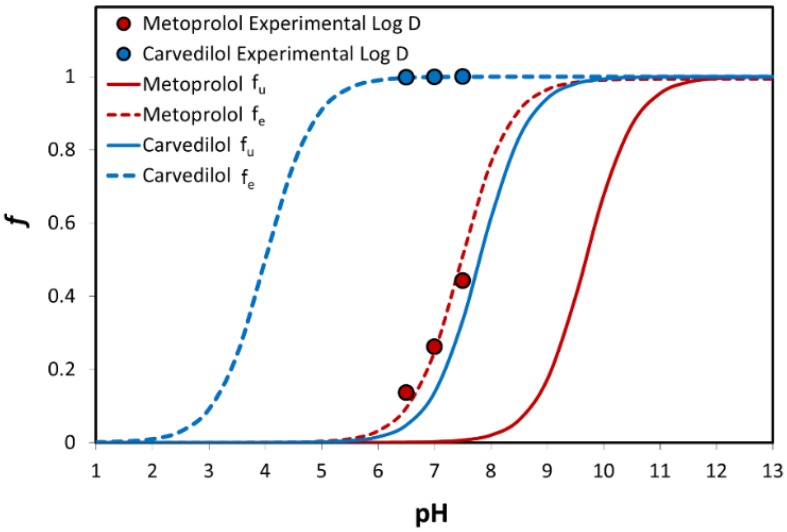
The theoretical fraction unionized (f_u_) and fraction extracted into octanol (f_e_) plots are presented as a function of pH for carvedilol and metoprolol. Log D values for both drugs are presented as circles; n = 6.

The theoretical fraction unionized (f_u_) and fraction extracted into octanol (f_e_) as a function of pH for carvedilol vs. metoprolol are presented in Figure 3. The f_u_ of the basic drugs carvedilol and metoprolol was negligible at low pH, and rose as the pH increased, producing a standard sigmoidal shape. It can be seen that the f_e_ vs. pH plot of both drugs shows a similar pattern, but with a shift to the left at the lower pH values. The shift degree equals to Log (P − 1) at the midpoint of the f_e_ and f_u_ sigmoidal curves [40]. Experimental octanol-buffer distribution of the drugs at the three pH values of 6.5, 7.0, and 7.5 are also presented in Figure 3 and are in excellent correlation with the theoretical plots.

### 3.5. PAMPA Assay

**Figure 4:**
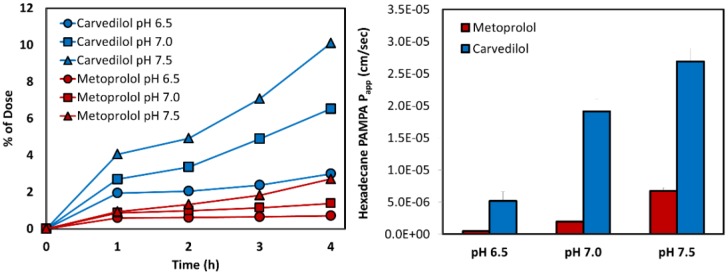
The hexadecane-based parallel artificial membrane permeability assay (PAMPA) permeability studies for carvedilol vs. metoprolol in the different pH conditions along the small intestine: amounts transported (mmol) as a function of time (left panel), and the corresponding P_app_ values (right panel; cm/s). Mean ± SD; n = 4.

The transported amounts vs. time in the PAMPA experiment for carvedilol and metoprolol are presented in Figure 4, with their matching P_app_ values. Compatibly to the log D results, the same pH-dependent upward permeability trend was found for both drugs; carvedilol showed considerably higher log D than metoprolol in the studied pH range, and the PAMPA permeability values confirmed this trend, as can be seen in Figure 4. 

### 3.6. Rat Intestinal Perfusion Studies

**Figure 5:**
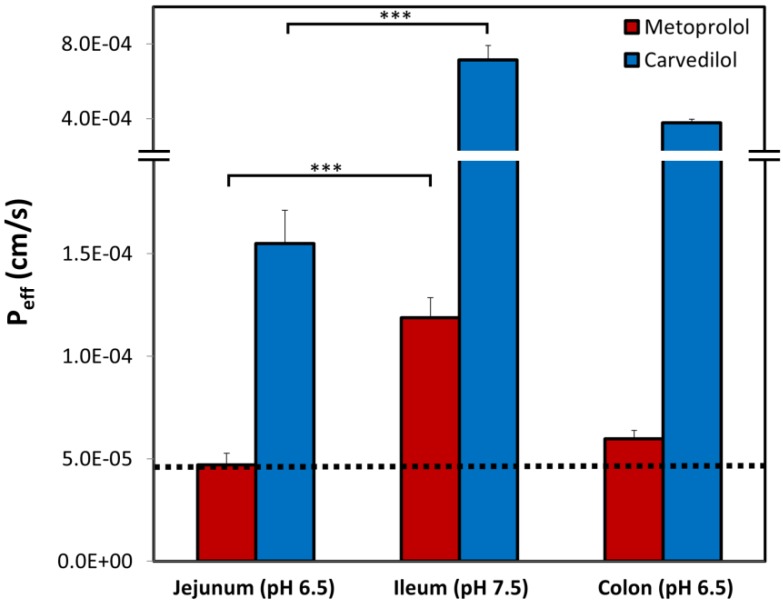
Effective permeability coefficient (P_eff_; cm/s) obtained for carvedilol vs. metoprolol in three rat intestinal segments, the upper jejunum (pH 6.5), terminal ileum (pH 7.5), and the colon (pH 6.5). The black dashed line represents the permeability of metoprolol in the jejunum (pH 6.5), which is the low/high P_eff_ class boundary standard. Data are presented as means ± SD; *** *p* < 0.001 between jejunum and ileum for both carvedilol and metoprolol; n = 6.

The values of carvedilol vs. metoprolol effective permeability coefficient (P_eff_) determined using the rat SPIP model, through the three intestinal segments: the proximal jejunum (pH 6.5), the distal ileum (pH 7.5), and the colon (pH 6.5), are presented in Figure 5. It can be observed that all of the permeability studies revealed a similar trend: higher pH led to higher permeability values, and as a result, the permeability of carvedilol and metoprolol in the ileum was significantly higher than in the jejunum. Furthermore, at any given intestinal segment/pH, the permeability of carvedilol was higher than that of metoprolol. In addition, when looking at the colon, the permeability value of carvedilol was higher than that of metoprolol in the jejunum (marked as a dashed line in Figure 5). 

## 4. Discussion

The variable physiological conditions throughout the GIT can greatly influence the rate and degree of oral drug absorption [10]. It was previously shown that there is a high level of correlation between the drug jejunal permeability and the fraction of dose absorbed from an IR drug product [11,47,48]. Conversely, for CR formulations, to obtain an optimal dissolution, intestinal permeability, and hence, acceptable bioavailability, a larger part of the GIT has to be accounted for in comparison to IR drug product, highlighting the crucial importance of regional variation among absorption factors. Both metoprolol and carvedilol are marketed as controlled-release products (metoprolol extended-release tablets of 25, 50, 100, or 200 mg; and carvedilol CR of 10, 20, 40, or 80 mg) which allows us to use them as model drugs in predicting important parameters that may dictate the development of a successful CR product [49,50].

Carvedilol is an alkaline drug that exhibits poor solubility in different conditions throughout the GIT [29]. However, the solubility studies revealed a high solubility at pH 4.0 (Table 1). In different studies, using simulated/aspirated media, it was shown that depending upon the experimental technique, some discrepancies are noticed [51,52], however, an apparent rise in solubility in the simulated intestinal fluid in the fed state (FeSSIF; pH = 5) is evident. This could be explained by the carvedilol chemical structure, where the aliphatic -NH group is more basic than the carbazole -NH group, which could lead to protonation, creating a soluble salt with the anionic form of the buffer, causing an increase in solubility [28,53]; in acidic media, the aliphatic -NH is ionized forming a cationic center, while in basic media, the carbazole -NH is ionized forming a anionic center. This zwitterionic nature is responsible for the unique solubility pattern presented in Table 1. At any rate, the low solubility values of carvedilol at acidic and neutral environment indicate a low-solubility BCS classification, as in the case of IR carvedilol product, a maximal single unit dose is 25 mg [23], leading to a dose number higher than 1 in different GIT locations. Alongside the high permeability values throughout the GIT, it was confirmed that carvedilol is indeed a BCS class II compound. 

Under such solubility limitations, developing carvedilol as a CR drug product may, in a way, help to avoid solubility limitations. By definition, a CR product releases the drug gradually from the formulation while traveling along the GIT, and so, in place of requiring the solubilization the entire dose at once, only a small fraction of the dose needs to be solubilized at a given time point, which may allow overcoming solubility limitations. On the other hand, at each point throughout the intestinal tract, the aqueous volume is lower than the 250 mL initially taken with the drug dose. In particular, it was reported that when simulating the fate of low-solubility drugs after oral administration, the small intestinal water volume that allowed the best fits with in vivo data was about 130 mL (ranging 10–150 mL in the fasted state), and 10 mL in the colon (with estimations as large as 125 mL in the fasted state) [54]. Nevertheless, if there is sufficient fluid in the lumen at each point, it may be possible to obtain adequate drug solubility throughout the digestive system. 

The pH-dilution dissolution experimental method mimics the passage and fate of the drug dose through the different GIT segments over time and hence, allows revealing whether the drug can be solubilized, and remain dissolved, while in the GIT. Our results elucidate that generally the highest IR dose (25 mg) has the ability to be dissolved, and remain such throughout the GIT travel, which explains why this formulation is an efficient marketed drug product. On the other hand, if the CR dose of carvedilol (80 mg) were to be administered at once as a simple IR formulation, rapid precipitation would take place (Figure 1), preventing the success of such drug products. Formulating this carvedilol dose as a CR product allows overcoming this solubility/dissolution limitation by distributing small portions of the dose at each stage throughout the GIT. This model analysis illustrates the application of biopharmaceutical aspects in the decision process of successful CR formulation development.

A literature search showed that carvedilol in vitro absorption was studied in a model called the Boehringer–Mannheim ring model using porcine intestine [55]. According to this study, the main route of absorption for carvedilol was transcellular, and the optimal absorption was obtained in the neutral pH of 6.8. In situ intestinal perfusion with mesenteric blood sampling in rats using human intestinal fluids and biorelevant media was used to study the food effect on the intestinal solubility and permeability of carvedilol [52]; however, the use of biorelevant media that contain high lecithin concentration would also affect the solubility aspect of carvedilol in comparison to our SPIP study. Therefore, we used the buffers for the perfusion study instead. This study also did not account for the segmental-dependency of the solubility/permeability of carvedilol.

Prior to evaluating intestinal permeability (P_eff_) results, the threshold for the low/high permeability class membership must be set, since it reflects the penetration degree that allows complete absorption. For this purpose, metoprolol is a widely used and accepted standard compound [56,57]. Metoprolol exhibits significant segmental-dependent intestinal permeability with increasing P_eff_ towards the distal parts of the small intestine. Therefore, the question is raised, which permeability should be taken as the class boundary: jejunal (~5 × 10^−5^ cm/s) or the much higher ileal value (~1.2 × 10^−4^ cm/s), presented in Figure 5. Absorption data obtained from humans revealed that 80% of metoprolol dose from IR product occurs already in the upper 50 cm of the small intestine (duodenum and proximal jejunum), leaving no drug for absorption in the ileum [58]. This was later shown in rats as well [59]. Therefore, ileal permeability values of metoprolol are not physiologically relevant for an IR drug product; it can be claimed that from an IR metoprolol product, no drug arrives into the ileum, as the entire dose gets absorbed much earlier. Hence, the jejunum permeability of metoprolol allows its complete absorption, and this value should be taken as the low/high threshold for permeability classification. Similarly, carvedilol demonstrated segmental-dependent permeability that matched the trend of metoprolol (the permeability in the ileum was significantly higher than in the jejunum, as demonstrated in Figure 5). However, the P_eff_ values of carvedilol were significantly higher than that of metoprolol in each intestinal segment. Importantly, colonic permeability values for both carvedilol and metoprolol were higher than that of metoprolol in the jejunum (illustrated as a dashed black line in Figure 5), validating the biopharmaceutical suitability of carvedilol, and metoprolol, to be developed as CR drug products. Typically, a CR drug product releases the drug continuously over 12–24, and since the transit time throughout the small intestine is 3-4 hours [60], the majority of the dose is released in the colon. This explains why high colonic drug permeability is a key biopharmaceutical factor in the decision process of CR drug product development. This permeability analysis of both model drugs (carvedilol and metoprolol) demonstrated the decision process required for successful CR dosage form development. 

Permeability studies both in vitro and in vivo (Log D, PAMPA, SPIP) resulted in higher permeability of carvedilol vs. metoprolol in all of the investigated segments/pHs. Both PAMPA and SPIP methods (Figure 4 and Figure 5) showed the same upward trend. The in vitro permeability models used in this work account for simple passive diffusion, without taking into account intestinal transporters, however, the in vivo SPIP model accounts for all permeability mechanisms, including active transport. When looking at the in vivo results (Figure 5), it can be seen that throughout the entire intestinal tract, carvedilol’s permeability was higher than that of metoprolol’s in the jejunum. In the colon (Figure 5), carvedilol’s permeability was higher than both metoprolol’s high/low permeability benchmark and permeability of carvedilol in the jejunum. The permeability of carvedilol in the colon was lower than in the ileum, likely due to a shift in the ionization state. This correlation between artificial and animal permeability studies depicts the main mechanism of carvedilol’s permeability as passive absorption. Furthermore, the octanol-buffer distribution coefficient of carvedilol was tremendously higher than that of metoprolol at different pH values (Figure 2). Even though Log P and Log D values are widely used as a replacement for passive intestinal permeability, relying solely on physicochemical drug properties when assessing drug permeation may lead to incorrect conclusions. For instance, the polar surface area (PSA) of carvedilol and metoprolol is 75.7 A^2^ and 50.7 A^2^ (Table 2), respectively [44,46]; lower PSA is usually associated with higher permeability, and hence, judging merely based on this characteristic would lead to the wrong conclusion. Therefore, prior to assigning a BCS classification, the many relevant aspects need to be thoroughly considered, to circumvent misconception in drug research, development, and regulation. 

The solubility–permeability interplay is an important part of evaluating a novel drug formulation. By merely looking at the solubility improvement that the formulation allows can be ambiguous in terms of predicting the consequent oral drug absorption, and vice versa, this interplay must be accounted for when aiming to optimize the solubility–permeability balance, and the overall drug absorption. Carvedilol is a low solubility compound whose solubility enhancement when developing a CR drug product relied on using a phosphate salt in the CR formulation. This increase in solubility might be responsible for a slight decrease in overall bioavailability. However, in the case of this drug product, it did not affect the clinical efficacy of carvedilol.

Carvedilol is both a substrate [26,61] and inhibitor [62] of the efflux transporter P-glycoprotein (P-gp). The involvement of intestinal transporters in general, and specifically P-gp, in the absorption process following oral administration is more biorelevant for low-permeability drugs, and the regional-dependent expression of the relevant transporters should be considered in these cases [63,64]. However, for high-permeability compounds, neither active uptake nor efflux transporters are expected to be rate-limiting [65,66]. Given that carvedilol has very high passive intestinal permeability throughout the entire GIT (Figure 5), the fact that it is a P-gp substrate would not be significant in the in vivo conditions. In addition, as mentioned before, carvedilol undergoes extensive stereoselective first-pass (CYP2D6 and CYP2C9). Similarly to transporters, intestinal enzymes may also exhibit regional-dependent expression, which needs to be accounted for when developing oral CR formulation [67]. Extensive knowledge of intestinal/hepatic transport and enzymatic metabolism is essential in the development process of a CR product.

## 5. Conclusions

Altogether, the analysis of carvedilol/metoprolol presented in this work serves as a model for a suitable candidate for a CR product development, from both the permeability and solubility/dissolution point of view. This work may increase our biopharmaceutical understanding of a successful CR drug product. 

Regional-dependent drug permeability and solubility/dissolution, and the effects of these factors on CR drug product development is often overlooked, and in this article, we aimed to emphasize these important issues; yet, additional data, including pharmacokinetics, metabolism, and pharmacotherapy considerations, are essential for the thorough prediction of a CR candidate. 

## Figures and Tables

**Table 1:** Carvedilol solubility values (mg/mL) in the tree pH values 1.0, 4.0, and 7.5, at 37 °C, and the corresponding dose number (D_0_) for a 25 mg dose. Data presented as mean ± SD; n = 6.

pH	Solubility (mg/mL)	Corresponding D_0_
1.0	0.021 (±0.004)	4.720
4.0	2.320 (±1.090)	0.043
7.5	0.035 (±0.002)	2.880

**Table 2:** Carvedilol and metoprolol molecular structures and relevant physicochemical parameters.

Drug	Molecular Structure	pKa	Log P	PSA
Carvedilol	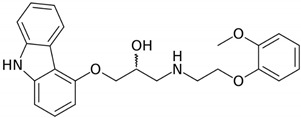	7.8 [43]	3.8 [43]	75.7 [44]
Metoprolol	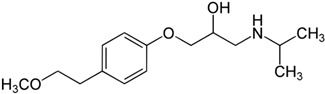	9.7 [42]	2.2 [45]	50.7 [46]

## References

[1] Dahan A., Miller J.M., Hilfinger J.M., Yamashita S., Yu L.X., Lennernas H., Amidon G.L. (2010). High-permeability criterion for BCS classification: Segmental/pH dependent permeability considerations. Mol. Pharm..

[2] Dahlgren D., Lennernas H. (2019). Intestinal Permeability and Drug Absorption: Predictive Experimental, Computational and In Vivo Approaches. Pharmaceutics.

[3] Fairstein M., Swissa R., Dahan A. (2013). Regional-dependent intestinal permeability and BCS classification: Elucidation of pH-related complexity in rats using pseudoephedrine. AAPS J..

[4] Amidon G.L., Lennernas H., Shah V.P., Crison J.R. (1995). A theoretical basis for a biopharmaceutic drug classification: The correlation of in vitro drug product dissolution and in vivo bioavailability. Pharm. Res..

[5] European Medicines Agency (2010). Guideline on the Investigation of Bioequivalence.

[6] U.S. Department of Health and Human Services, Food and Drug Administration, Center for Drug Evaluation and Research (2017). Waiver of In-Vivo Bioavailability and Bioequivalence Studies for Immediate-Release Solid Oral Dosage Forms Based on a Biopharmaceutics Classification System. Guidance for Industry.

[7] Garcia-Arieta A., Gordon J. (2012). Bioequivalence requirements in the European Union: Critical discussion. AAPS J..

[8] Dahan A., Wolk O., Zur M., Amidon G.L., Abrahamsson B., Cristofoletti R., Groot D.W., Kopp S., Langguth P., Polli J.E. (2014). Biowaiver Monographs for Immediate-Release Solid Oral Dosage Forms: Codeine Phosphate. J. Pharm. Sci..

[9] Ozawa M., Tsume Y., Zur M., Dahan A., Amidon G.L. (2015). Intestinal permeability study of minoxidil: Assessment of minoxidil as a high permeability reference drug for biopharmaceutics classification. Mol. Pharm..

[10] Dahan A., Lennernäs H., Amidon G.L. (2012). The Fraction Dose Absorbed, in Humans, and High Jejunal Human Permeability Relationship. Mol. Pharm..

[11] Lennernas H. (2014). Regional intestinal drug permeation: Biopharmaceutics and drug development. Eur. J. Pharm. Sci..

[12] Zur M., Gasparini M., Wolk O., Amidon G.L., Dahan A. (2014). The Low/High BCS Permeability Class Boundary: Physicochemical Comparison of Metoprolol and Labetalol. Mol. Pharm..

[13] Zur M., Hanson A.S., Dahan A. (2014). The complexity of intestinal permeability: Assigning the correct BCS classification through careful data interpretation. Eur. J. Pharm. Sci.

[14] Dahan A., Sabit H., Amidon G.L. (2009). The H2 receptor antagonist nizatidine is a P-glycoprotein substrate: Characterization of its intestinal epithelial cell efflux transport. AAPS J..

[15] Lennernas H. (2014). Human in vivo regional intestinal permeability: Importance for pharmaceutical drug development. Mol. Pharm..

[16] Ungell A.L., Nylander S., Bergstrand S., Sjoberg A., Lennernas H. (1998). Membrane transport of drugs in different regions of the intestinal tract of the rat. J. Pharm. Sci..

[17] Corrigan O.I. (1997). The biopharmaceutic drug classification and drugs administered in extended release (ER) formulations. Adv. Exp. Med. Biol..

[18] Tannergren C., Bergendal A., Lennernas H., Abrahamsson B. (2009). Toward an increased understanding of the barriers to colonic drug absorption in humans: Implications for early controlled release candidate assessment. Mol. Pharm..

[19] Xu J., Lin Y., Boulas P., Peterson M.L. (2018). Low colonic absorption drugs: Risks and opportunities in the development of oral extended release products. Expert Opin. Drug Deliv..

[20] Frishman W.H. (1998). Carvedilol. N. Engl. J. Med..

[21] Kukin M.L. (2002). β-Blockers in Chronic Heart Failure: Considerations for Selecting an Agent. Mayo Clin. Proc..

[22] Henderson L.S., Tenero D.M., Baidoo C.A., Campanile A.M., Harter A.H., Boyle D., Danoff T.M. (2006). Pharmacokinetic and pharmacodynamic comparison of controlled-release carvedilol and immediate-release carvedilol at steady state in patients with hypertension. Am. J. Cardiol..

[23] Packer M., Lukas M.A., Tenero D.M., Baidoo C.A., Greenberg B.H. (2006). Pharmacokinetic profile of controlled-release carvedilol in patients with left ventricular dysfunction associated with chronic heart failure or after myocardial infarction. Am. J. Cardiol.

[24] Incecayir T., Tsume Y., Amidon G.L. (2013). Comparison of the permeability of metoprolol and labetalol in rat, mouse, and Caco-2 cells: Use as a reference standard for BCS classification. Mol. Pharm..

[25] Brodde O.E., Kroemer H.K. (2003). Drug-drug interactions of beta-adrenoceptor blockers. Arzneim. Forsch..

[26] Baris N., Kalkan S., Guneri S., Bozdemir V., Guven H. (2006). Influence of carvedilol on serum digoxin levels in heart failure: Is there any gender difference?. Eur. J. Clin. Pharmacol..

[27] Giessmann T., Modess C., Hecker U., Zschiesche M., Dazert P., Kunert-Keil C., Warzok R., Engel G., Weitschies W., Cascorbi I. (2004). CYP2D6 genotype and induction of intestinal drug transporters by rifampin predict presystemic clearance of carvedilol in healthy subjects. Clin. Pharmacol. Ther..

[28] Hamed R., Awadallah A., Sunoqrot S., Tarawneh O., Nazzal S., AlBaraghthi T., Al Sayyad J., Abbas A. (2016). pH-Dependent Solubility and Dissolution Behavior of Carvedilol-Case Example of a Weakly Basic BCS Class II Drug. AAPS PharmSciTech.

[29] Varma M.V., Gardner I., Steyn S.J., Nkansah P., Rotter C.J., Whitney-Pickett C., Zhang H., Di L., Cram M., Fenner K.S. (2012). pH-Dependent Solubility and Permeability Criteria for Provisional Biopharmaceutics Classification (BCS and BDDCS) in Early Drug Discovery. Mol. Pharm..

[30] Zur M., Cohen N., Agbaria R., Dahan A. (2015). The biopharmaceutics of successful controlled release drug product: Segmental-dependent permeability of glipizide vs. metoprolol throughout the intestinal tract. Int. J. Pharm..

[31] Beig A., Miller J.M., Lindley D., Dahan A. (2017). Striking the Optimal Solubility-Permeability Balance in Oral Formulation Development for Lipophilic Drugs: Maximizing Carbamazepine Blood Levels. Mol. Pharm..

[32] Fine-Shamir N., Beig A., Zur M., Lindley D., Miller J.M., Dahan A. (2017). Toward Successful Cyclodextrin Based Solubility-Enabling Formulations for Oral Delivery of Lipophilic Drugs: Solubility–Permeability Trade-Off, Biorelevant Dissolution, and the Unstirred Water Layer. Mol. Pharm..

[33] Fine-Shamir N., Dahan A. (2019). Methacrylate-Copolymer Eudragit EPO as a Solubility-Enabling Excipient for Anionic Drugs: Investigation of Drug Solubility, Intestinal Permeability, and Their Interplay. Mol. Pharm..

[34] Dahan A., West B.T., Amidon G.L. (2009). Segmental-dependent membrane permeability along the intestine following oral drug administration: Evaluation of a triple single-pass intestinal perfusion (TSPIP) approach in the rat. Eur. J. Pharm. Sci..

[35] Lozoya-Agullo I., Zur M., Fine-Shamir N., Markovic M., Cohen Y., Porat D., Gonzalez-Alvarez I., Gonzalez-Alvarez M., Merino-Sanjuan M., Bermejo M. (2017). Investigating drug absorption from the colon: Single-pass vs. Doluisio approaches to in-situ rat large-intestinal perfusion. Int. J. Pharm..

[36] Wolk O., Markovic M., Porat D., Fine-Shamir N., Zur M., Beig A., Dahan A. (2019). Segmental-Dependent Intestinal Drug Permeability: Development and Model Validation of In Silico Predictions Guided by In Vivo Permeability Values. J. Pharm. Sci..

[37] Lozoya-Agullo I., Zur M., Beig A., Fine N., Cohen Y., Gonzalez-Alvarez M., Merino-Sanjuan M., Gonzalez-Alvarez I., Bermejo M., Dahan A. (2016). Segmental-dependent permeability throughout the small intestine following oral drug administration: Single-pass vs. Doluisio approach to in-situ rat perfusion. Int. J. Pharm..

[38] Lozoya-Agullo I., Zur M., Wolk O., Beig A., Gonzalez-Alvarez I., Gonzalez-Alvarez M., Merino-Sanjuan M., Bermejo M., Dahan A. (2015). In-situ intestinal rat perfusions for human Fabs prediction and BCS permeability class determination: Investigation of the single-pass vs. the Doluisio experimental approaches. Int. J. Pharm..

[39] Tugcu-Demiroz F., Gonzalez-Alvarez I., Gonzalez-Alvarez M., Bermejo M. (2014). Validation of phenol red versus gravimetric method for water reabsorption correction and study of gender differences in Doluisio’s absorption technique. Eur. J. Pharm. Sci..

[40] Wagner J.G., Sedman A.J. (1973). Quantitaton of rate of gastrointestinal and buccal absorption of acidic and basic drugs based on extraction theory. J. Pharmacokinet. Biopharm..

[41] Winne D. (1977). Shift of pH-absorption curves. J. Pharmacokinet. Biopharm..

[42] Teksin Z.S., Hom K., Balakrishnan A., Polli J.E. (2006). Ion pair-mediated transport of metoprolol across a three lipid-component PAMPA system. J. Control. Release.

[43] Tsume Y., Mudie D.M., Langguth P., Amidon G.E., Amidon G.L. (2014). The Biopharmaceutics Classification System: Subclasses for in vivo predictive dissolution (IPD) methodology and IVIVC. Eur. J. Pharm. Sci..

[44] National Center for Biotechnology Information PubChem Database. Carvedilol, CID=2585. https://pubchem.ncbi.nlm.nih.gov/compound/Carvedilol.

[45] Henchoz Y., Guillarme D., Martel S., Rudaz S., Veuthey J.-L., Carrupt P.-A. (2009). Fast log P determination by ultra-high-pressure liquid chromatography coupled with UV and mass spectrometry detections. Anal. Bioanal. Chem..

[46] National Center for Biotechnology Information PubChem Database. Metoprolol, CID=4171. https://pubchem.ncbi.nlm.nih.gov/compound/Metoprolol.

[47] Lennernas H. (1998). Human intestinal permeability. J. Pharm. Sci..

[48] Lennernas H. (2007). Animal data: The contributions of the Ussing Chamber and perfusion systems to predicting human oral drug delivery in vivo. Adv. Drug Deliv. Rev..

[49] COREG CR™ (Carvedilol Phosphate) Extended-Release Capsules Prescribing Information. https://www.accessdata.fda.gov/drugsatfda_docs/label/2007/022012s003lbl.pdf.

[50] Metoprolol SuccinateTM Extended-Release Tablets, Prescribing Information. https://www.accessdata.fda.gov/drugsatfda_docs/label/2006/019962s032lbl.pdf.

[51] Fagerberg J.H., Tsinman O., Sun N., Tsinman K., Avdeef A., Bergström C.A.S. (2010). Dissolution rate and apparent solubility of poorly soluble drugs in biorelevant dissolution media. Mol. Pharm..

[52] Stappaerts J., Wuyts B., Tack J., Annaert P., Augustijns P. (2014). Human and simulated intestinal fluids as solvent systems to explore food effects on intestinal solubility and permeability. Eur. J. Pharm. Sci..

[53] Loftsson T., Vogensen S.B., Desbos C., Jansook P. (2008). Carvedilol: Solubilization and cyclodextrin complexation: A technical note. AAPS PharmSciTech.

[54] Sutton S.C. (2009). Role of physiological intestinal water in oral absorption. AAPS J..

[55] Nolte K., Backfisch G., Neidlein R. (1999). In vitro absorption studies with carvedilol using a new model with porcine intestine called BM-RIMO (Boehringer-Mannheim ring model). Arzneim. Forsch..

[56] Dahan A., Miller J.M., Amidon G.L. (2009). Prediction of solubility and permeability class membership: Provisional BCS classification of the world’s top oral drugs. AAPS J..

[57] Kim J.S., Mitchell S., Kijek P., Tsume Y., Hilfinger J., Amidon G.L. (2006). The suitability of an in situ perfusion model for permeability determinations: Utility for BCS class I biowaiver requests. Mol. Pharm..

[58] Jobin G., Cortot A., Godbillon J., Duval M., Schoeller J.P., Hirtz J., Bernier J.J. (1985). Investigation of drug absorption from the gastrointestinal tract of man. I. Metoprolol in the stomach, duodenum and jejunum. Br. J. Clin. Pharmacol..

[59] Masaoka Y., Tanaka Y., Kataoka M., Sakuma S., Yamashita S. (2006). Site of drug absorption after oral administration: Assessment of membrane permeability and luminal concentration of drugs in each segment of gastrointestinal tract. Eur. J. Pharm. Sci..

[60] Davis S.S., Hardy J.G., Fara J.W. (1986). Transit of pharmaceutical dosage forms through the small intestine. Gut.

[61] Bart J., Dijkers E.C.F., Wegman T.D., de Vries E.G.E., van der Graaf W.T.A., Groen H.J.M., Vaalburg W., Willemsen A.T.M., Hendrikse N.H. (2005). New positron emission tomography tracer [11C] carvedilol reveals P-glycoprotein modulation kinetics. Br. J. Clin. Pharmacol..

[62] Wessler J.D., Grip L.T., Mendell J., Giugliano R.P. (2013). The P-glycoprotein transport system and cardiovascular drugs. J. Am. Coll. Cardiol..

[63] Dahan A., Sabit H., Amidon G.L. (2009). Multiple Efflux Pumps Are Involved in the Transepithelial Transport of Colchicine: Combined Effect of P-Glycoprotein and Multidrug Resistance-Associated Protein 2 Leads to Decreased Intestinal Absorption Throughout the Entire Small Intestine. Drug Metab. Dispos..

[64] MacLean C., Moenning U., Reichel A., Fricker G. (2008). Closing the Gaps: A Full Scan of the Intestinal Expression of P-Glycoprotein, Breast Cancer Resistance Protein, and Multidrug Resistance-Associated Protein 2 in Male and Female Rats. Drug Metab. Dispos..

[65] Dahan A., Amidon G.L. (2008). Segmental Dependent Transport of Low Permeability Compounds along the Small Intestine Due to P-Glycoprotein: The Role of Efflux Transport in the Oral Absorption of BCS Class III Drugs. Mol. Pharm..

[66] Giacomini K.M., Huang S.-M., Tweedie D.J., Benet L., Brouwer K.L., Chu X., Dahlin A., Evers R., Fischer V., International Transporter Consortium (2010). Membrane transporters in drug development. Nat. Rev. Drug Discov..

[67] Tubic-Grozdanis M., Hilfinger J.M., Amidon G.L., Kim J.S., Kijek P., Staubach P., Langguth P. (2008). Pharmacokinetics of the CYP 3A Substrate Simvastatin following Administration of Delayed Versus Immediate Release Oral Dosage Forms. Pharm. Res..

